# From Crisis to Control: A Study of Typhoid Conjugate Vaccine Efficacy in Harare, Zimbabwe (2017–2024)

**DOI:** 10.1093/ofid/ofag091

**Published:** 2026-03-16

**Authors:** Talent Bvochora, John Manyara, Gaetan Thilliez, Michael Vere, Innocent Mukeredzi, Denford Nhamo, Farai Chitiyo, Augustine Muzondo, Agnes Juru, Prosper Chonzi, Isaac Phiri, Anthony M Smith, Blessmore V Chaibva, Munyaradzi Mapingure, Walter Fuller, Pramila Shrestha, Parvati Nair, Robert A Kingsley, Ramanan Laxminarayan, Godfrey Musuka, Tapfumanei Mashe

**Affiliations:** Harare City Health Department, Harare City Municipality, Harare, Zimbabwe; Harare City Health Department, Harare City Municipality, Harare, Zimbabwe; School of Biotechnology, Dublin City University, Dublin, Ireland; Harare City Health Department, Harare City Municipality, Harare, Zimbabwe; Harare City Health Department, Harare City Municipality, Harare, Zimbabwe; Harare City Health Department, Harare City Municipality, Harare, Zimbabwe; Harare Metropolitan Province, Medical Directorate, Harare, Zimbabwe; National Microbiology Reference Laboratory, Ministry of Health and Child Care, Harare, Zimbabwe; National Microbiology Reference Laboratory, Ministry of Health and Child Care, Harare, Zimbabwe; Harare City Health Department, Harare City Municipality, Harare, Zimbabwe; Division of the National Health Laboratory Service, Epidemiology and Disease Control, Ministry of Health and Child Care, Harare, Zimbabwe; Centre for Enteric Diseases, National Institute for Communicable Diseases, Johannesburg, South Africa; Department of Medical Microbiology, Faculty of Health Sciences, University of Pretoria, Pretoria, South Africa; Department of Pharmacy, Ministry of Health and Child Care, Harare, Zimbabwe; Department of Global Public Health and Family Medicine, Faculty of Medicine and Health Sciences, University of Zimbabwe, Harare, Zimbabwe; Antimicrobial Resistance Unit, Health Systems and Services Cluster, WHO Regional Office for Africa (AFRO), Cité du Djoué, Brazzaville, Republic of Congo; Antimicrobial Resistance Department, World Health Organization, Geneva, Switzerland; Global Health and Humanitarian Medicine, Médecins Sans Frontières (MSF) South Asia, Colombo, Sri Lanka; Microbes and Food Safety Department, Quadram Institute Bioscience, Norwich, UK; School of Biological Sciences, University of East Anglia, Norwich, UK; Founder and President, One Health Trust, Bengaluru, India; High Meadows Environmental Institute, Princeton University, Princeton, New Jersey, USA; 3ieimpact, Harare, Zimbabwe; Health Systems Strengthening Unit, World Health Organization, Harare, Zimbabwe

**Keywords:** antimicrobial resistance, attack rates, effectiveness, Harare, typhoid vaccination

## Abstract

**Background:**

Typhoid fever remains a public health concern in Harare City, Zimbabwe. Recurrent outbreaks are driven by inadequate water, sanitation, and hygiene infrastructure. In 2019, the typhoid conjugate vaccine (TCV) was introduced. The TCV impact on typhoid epidemiology, antimicrobial resistance (AMR), *Salmonella* Typhi population, and effectiveness across districts and age groups remains understudied.

**Methods:**

Data from 3401 typhoid cases during 2017–2024 were analyzed. Attack rates, risk ratios, AMR, and vaccine effectiveness across prevaccine (2017–2019) and postvaccine (2020–2024) periods were compared. Analysis was stratified by district, vaccination coverage, and age groups. Genomic characteristics of *Salmonella* Typhi strains isolated postvaccination were investigated and compared to prevaccine populations.

**Results:**

Attack rates for the Western district, which reported 70.8% of cases, decreased from 1373/100 000 before TCV to 341/100 000 after (risk ratio: 0.40, *P* ≤ .0001). Subdistricts had attack rates of 1783 (Glen View), 1687 (Mufakose), and 1145 (Budiriro) per 100 000 before vaccination and 223, 33, and 364/100 000, respectively, after (risk ratio: 0.22, 0.03, 0.48, respectively, *P* < .0001). The 0–15 age group showed vaccine effectiveness of 81.2% (95% confidence interval, 71.2–88.8), compared to 61.4% (95% confidence interval, 54.3–68.1) across all ages. Genomic comparison of *Salmonella* Typhi isolates pre- and postvaccination did not indicate changes in bacterial population. AMR phenotypic data and genomic prediction indicated lower resistance to antibiotics postvaccination.

**Conclusions:**

TCV reduced typhoid incidence, particularly in high-burden areas and children. No shift in the *Salmonella* Typhi population was observed. Ongoing transmission underscores need for integrated measures, including human-resource capacity, improved water, sanitation, and hygiene infrastructure, research on vaccine performance variability, and refined multisectoral interventions.

Typhoid fever, an enteric disease caused by the bacterium *Salmonella enterica* serovar Typhi (*Salmonella* Typhi), infects 9 million people and causes 110 000 deaths annually [1]. Globally, *Salmonella* Typhi caused 76.3% of enteric fever cases with a case fatality of 0.95% in 2017 [2]. Higher case fatality estimates have been reported among children and older adults, and among those living in low-income countries [3], where inadequate water, sanitation, and hygiene (WASH) infrastructure facilitates transmission through contaminated food and water [4].

Sub-Saharan Africa experiences a disproportionately higher burden of disease at 762 cases per 100 000 person-years, significantly higher than in Southeast Asia, East Asia, and Oceania, with 108 per 100 000 person-years [5]. Key drivers include high population density, poor socioeconomic status, and poor access to WASH infrastructure. Although typhoid is treatable with antibiotics, the rise of multidrug resistant strains over the past decade has significantly complicated its management, reinforcing the need for preventive strategies [6].

Vaccination may represent the most effective strategy for typhoid control in the short to medium term, and long-term approach must include improvement of WASH infrastructure and practices [7]. Recommended by the World Health Organization for endemic regions, the typhoid conjugate vaccine (TCV) offers long-lasting immunity and is safe for children as young as 6 months of age [8]. Its integration into routine immunization programs has shown promise in reducing the burden of typhoid fever in high-risk settings [9]. Several countries including Malawi, Pakistan, Liberia, Zimbabwe, and Nepal have introduced TCV into routine immunization, significantly reducing disease burden [7].

Typhoid fever remains a pressing public health problem in Harare, Zimbabwe, with recurrent outbreaks causing substantial morbidity. Between August 2018 and February 2019, Harare recorded 1967 suspected and confirmed typhoid cases [10]. The Western suburbs, such as Glenview and Budiriro, have consistently reported the highest burden of cases, accounting for >50% of the city's total cases during outbreaks [11]. These outbreaks are driven by poor WASH infrastructure, compounded by reliance on contaminated boreholes [11]. In response, the Ministry of Health and Child Care introduced TCV in February 2019 [12]. Despite the introduction of the TCV in 2019, cases persisted, with an estimated vaccine effectiveness of 67% among children aged 0–15 years [13].

Evaluating epidemiological trends and vaccine effectiveness will provide critical insights to help refine vaccination strategies, enhance public health interventions, and strengthen disease surveillance. This retrospective review describes the epidemiology of typhoid outbreaks in selected suburbs of Harare City from 2017 to 2024 and analyses the attack rates and effectiveness of vaccination. Genomic analysis of *Salmonella* Typhi isolates was also conducted to complement the phenotypic antimicrobial resistance (AMR) data and assess whether the vaccination campaign acted as a selection pressure, resulting in a shift in the bacterial population causing typhoid. The findings could help fine-tune the use of TCV in typhoid control programs in resource-limited settings.

## MATERIALS AND METHODS

### Study Design

A pre- and postcommunity intervention comparative study.

### Definitions

#### Suspect (Suspected) Typhoid Case

A patient with sustained fever of ≥38 °C lasting for at least 3 consecutive days and the presence of ≥1 of the following symptoms: headache, abdominal pain or discomfort, weakness and malaise, loss of appetite, and diarrhea or constipation.

#### Positive (Confirmed) Typhoid Case

A patient who presents with clinical signs and symptoms consistent with typhoid fever (as mentioned previously) and whose diagnosis is verified through laboratory testing, by isolating *Salmonella* Typhi from a blood or stool culture.

#### Negative Typhoid Case

A patient, initially suspected of having typhoid based on clinical presentation, is subsequently determined not to be infected with *Salmonella* Typhi based on laboratory testing.

#### Attack Rate

The measure used to describe the frequency of new cases of a disease in a specific population over a defined period, particularly during an outbreak. It helps identify high-risk areas and populations, guiding targeted public health interventions.


$$\begin{array}{r} \mathrm{Attack}\;\mathrm{Rate}=\frac{\mathrm{Number}\;\mathrm{of}\;\mathrm{new}\;\mathrm{cases}\;\mathrm{during}\;\mathrm{the}\;\mathrm{period}}{\mathrm{Population}\;\;\mathrm{at}\;\mathrm{risk}\;\mathrm{during}\;\mathrm{the}\;\mathrm{same}\;\mathrm{period}}\times 1000 \end{array}$$

### Study Setting

This study was conducted in Harare City, the capital of Zimbabwe, with a population of 1 846 437 according to the 2022 census by ZIMSTAT [14]. Harare City has 4 districts: Southern, Eastern, Western, and Northern. Health services are provided through 2 infectious diseases hospitals (Wilkins and Beatrice Road), an emergency services center, and 43 clinics, which are distributed across its 4 districts. Nine of 27 residential areas of Harare City were typhoid hotspots, namely Mbare, Hopley, Mufakose, Glenview, Glenorah, Budiriro, Dzivarasekwa, Kuwadzana, and Hatcliffe, and where the TCV campaign was rolled out in 2019. The study used data collected from these residential areas.

### Study Population

The study used the Harare City typhoid records (2017–2024), which includes all reported cases across the aforementioned residential areas. Facility-level data were reported and consolidated centrally into a line list and these are the data analyzed. No cases were reported from 2020–2021 from the facilities, and analyses were restricted to periods whose surveillance data was reported. Data variables included age, sex, residence, and vaccination status.

#### Inclusion Criteria

All suspected and confirmed typhoid cases between 2017 and 2024 residing in the 9 Harare City suburbs mentioned previously.

#### Exclusion Criteria

Cases from outside these 9 Harare City suburbs.

#### Sample Size

A total of 3401 typhoid cases were included.

#### Data Collection and Analysis

Data were extracted electronically from the line list, cleaned, and analyzed by Stata V.18.5. Descriptive statistics using frequencies and proportions were calculated. Typhoid attack rates and the relative risk before and after vaccination were also calculated. Vaccine effectiveness was also calculated for 0- to 15-year-olds and all age groups.

### *Salmonella* Typhi Isolates and Transportation

A total of 26 postvaccination *Salmonella* Typhi were collected from Beatrice Road Infectious Diseases Hospital, which is a referral laboratory for all 52 clinics in Harare. The isolates were shipped to South Africa, according to IATA UN3373 regulations. All shipments were conducted under a formal Material Transfer Agreement and accompanied by the required import permits from the South African Department of Health to maintain a secure and compliant chain of custody.

### DNA Extraction and Sequencing

The platform used for library preparation, sequencing, and accession number was at the National Institute for Communicable Diseases, South Africa, and whole-genome sequencing of post-TCV isolates was performed as follows. Genomic DNA was extracted from bacterial cultures using the Invitrogen PureLink Microbiome DNA Purification Kit (Invitrogen, Waltham, Massachusetts, USA). Whole-genome sequencing was performed using the Illumina NextSeq 2000 (Illumina, San Diego, California, USA), with DNA libraries prepared using the Illumina DNA Prep Kit (Illumina), followed by 2 × 150-bp paired-end sequencing runs with ∼80 times coverage.

Genomic analysis of the genomes was carried out as described in the supplemental methods (Supplementary Table 1).

### Permission and Ethical Considerations

Permission to carry out the study was obtained from the Harare City institutional review board. Patient data were available only to investigators and confidentiality was maintained.

## RESULTS

A total of 3401 cases were studied spanning the period 2017–2024. Before the TCV campaign (2017–2019), there were 2521 cases and after the TCV campaign (2020–2024), there were 880 cases, with no cases reported in Harare City records for 2021. Of the 880 post-TCV, 339 had an unknown vaccination status, 415 were not vaccinated, and 126 were vaccinated.

Table 1 represents the demographic and clinical characteristics of typhoid cases in Harare before (2017–2019) and after (2020–2024) the introduction of TCV. The gender distribution was consistent, with males accounting for more cases in both periods. The cases among children younger than 5 years before and after TCV deployment did not show significant change, whereas cases between 5 and 15 years showed substantial reduction, with 52% pre- and 19% post-TCV. In contrast, those aged 16–45 years had 26% of cases before TCV and 56% of cases post-TCV. Suspected cases were 97% before TCV and 64% after TCV, indicating a considerable impact, but positive cases were 2% and 5%, before and after TCV. Of the cases after vaccination, 15% had received the vaccine, whereas 47% were not vaccinated and 38% had unknown vaccination status. The Western district reported 76% of the cases before TCV and 54% of the cases after TCV, pointing to a considerable reduction in morbidity.

**Table 1.** Demographic Characteristics of Typhoid Cases Before (2017–2019) and After (2020–2024) TCV in Harare City

Variable	Category	2017–2019, n (%)	2020–2024, n (%)
Sex	Male	1309 (52)	461 (52)
	Female	1202 (48)	419 (48)
Age in years	<5	665 (22)	217 (25)
	5–15	554 (52)	172 (19)
	>15	1302 (26)	491 (56)
Median age in years	(IQR)	17 (4–29)	19 (5–34)
Case status	Suspects	2443 (97)	558 (64)
	Positive	61 (2)	46 (5)
	Negative	17 (1)	276 (31)
Vaccination status	No vaccine	2521 (100)	…
	Unknown	…	339 (38)
	Not vaccinated	…	412 (47)
	Vaccinated	…	129 (15)
Clinical presentation	Fever (Temperature >38 °C)	1745 (69)	786 (89)
	Diarrhea	1383 (55)	549 (62)
	Headache	621 (25)	405 (46)
	Abdominal pain	629 (25)	249 (28)
	Vomiting	674 (27)	199 (23)
	Myalgia	189 (8)	123 (14)
	Constipation	60 (2)	24 (3)
	Loss of appetite	9 (1)	18 (2)
District of residence	Western	1930 (76)	479 (54)
	Southern	394 (16)	327 (37)
	Northern	176 (7)	60 (7)
	Eastern	21 (1)	14 (2)

Abbreviation: TCV, typhoid conjugate vaccine.

### Epicurve of Typhoid Cases From 2017 to 2024 in Harare City

**Figure 1.**
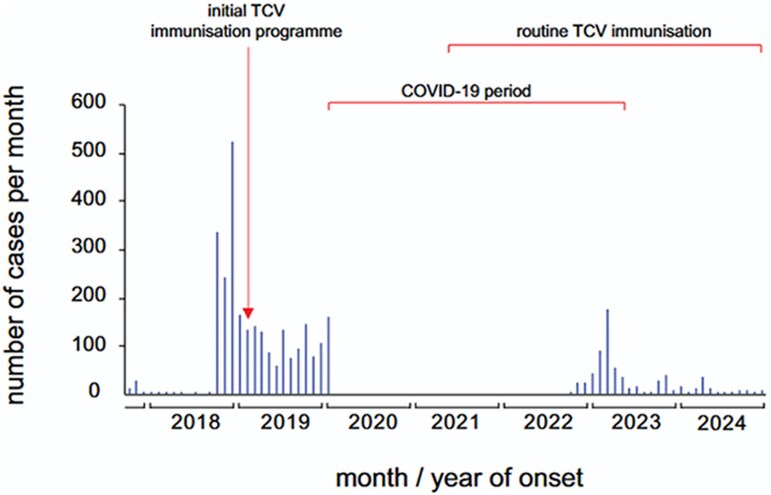
Epicurve of typhoid cases from 2017 to 2024 in Harare City.

The epidemic curve, Figure 1, shows cases by months of onset from 2017 to 2024. The first case in 2017 was recorded on 27 October 2017. The highest peak of cases was recorded in December 2018. Mass vaccination started on 15 February 2019, targeting children aged 6 months to 15 years, and was done for 2 weeks. Routine vaccination of children at 9 months of age started in May 2021. Almost three quarters of the cases, 2521/3401 (74%), were reported before TCV. Cases continued to increase progressively, suggesting a propagated outbreak.

### Attack Rates of *Salmonella* Typhi Infections by Districts Before (2017–2019) and After (2020–2024) TCV in Harare City

Of the 4 districts, the Western district, the attack rate was 1373/100 000 pre-TCV (2017–2019) and 341/100 000 post-TCV (2020–2024), with a risk ratio of 0.40 (95% confidence interval [CI], .37–.43; *P* < .0001). The Southern district and Northern district also had significant differences in attack rates pre- and post-TCV (*P* < .0001), as shown in Table 2. The Eastern district attack was 120 and 40/100,000, showing a risk ratio of 0.80 (95% CI, .53–1.20) and a *P* value of .24, indicating no statistical significance.

**Table 2.** Attack Rates of *Salmonella* Typhi Infections by Districts Before (2017–2019) and After (2020–2024) TCV in Harare City

District	Estimated Population	Cases		Attack Rate/100 000		Risk Ratio	95% CI	*P* Value
		2017–2019	2020–2024	2017–2019	2020–2024		…	…
Western	140 534	1930	479	1373	341	0.40	.37–.43	<.0001
Southern	125 293	394	327	314	261	0.91	.84–.98	.01
Northern	89 667	176	60	196	67	0.51	.41–.63	<.0001
Eastern	17 533	21	14	120	40	0.80	.53–1.20	.24

Abbreviations: CI, confidence interval; TCV, typhoid conjugate vaccine.

### Attack Rates of Typhoid Before (2017–2019) and After (2020–2024) TCV in Areas that Received the Vaccine, Harare City

In Harare City, 9 areas (depicted in Table 3) received TCV during the 2019 campaign. The attack rates for most areas declined (with *P* values indicative of statistical significance), specifically Mufakose, Glenview, Budiriro, and Dzivaresekwa except Glen Noral (*P* = .142) and Hopley.

**Table 3.** Attack Rates of Typhoid Before (2017–2019) and After (2020–2024) TCV in Areas that Received the Vaccine, Harare City

Area	Estimated Vaccinated Population	Cases		Attack Rate/100 000		Risk Ratio	95% CI	*P* Value
		2017–2019	2020–2024	2017–2019	2020–2024			
Glenview	43 961	784	98	1783	223	0.22	.20–.25	<.0001
Mufakose	21 446	362	7	1687	33	0.03	.03–.05	<.0001
Budiriro	46 876	537	171	1145	364	0.48	.44–.52	.01
Glen Norah	28 251	130	143	460	506	1.05	.99–1.11	.14
Dzivaresekwa	27 399	96	27	350	98	0.44	.37–.52	<.0001
Hopley	43 924	139	240	316	546	127	1.20–1.33	<.0001
Mbare	81 369	255	91	313	112	0.52	.45	<.0001
Hatcliffe	17 533	21	14	120	80	0.80	.67–.95	.01
Kuwadzana	62 268	64	33	103	53	0.67	.55–.85	<.0001

Abbreviations: CI, confidence interval; TCV, typhoid conjugate vaccine.

### Age-specific Attack Rates of *Salmonella* Typhi Infections in Harare City, Before, 2017–2019 and After TCV, 2020–2024

Among children aged 0–4 years, the attack rate was 2225/100 000 before TCV implementation (2017–2019) and 726/100 000 after (2020–2024), with a risk ratio of 0.25 (95% CI, .22–.28; *P* < .0001). All other age groups, aged 5–15 years, and older than 16 years also showed statistically significant differences in attack rates pre- and post-TCV as shown in Table 4.

**Table 4.** Age-specific Attack Rates of *Salmonella* Typhi Infections in Harare City, 2017 to 2024

Age Group	Estimated Population	Cases		Attack Rate/100 000		Risk Ratio	95% CI	*P* Value
		2017–2019	2020–2024	2017–2019	2020–2024			
0–4	29 888	665	217	2225	726	0.25	.22–.28	<.0001
5–15	77 536	554	172	715	222	0.26	.23–.29	<.0001
>15	234 030	1302	491	556	210	0.23	.21–.25	<.0001

Abbreviation: CI, confidence interval.

### Vaccine Effectiveness (Direct) for Harare City, 2020–2024

For all age groups, vaccination was associated with a 61.4% reduction in the risk of testing positive for typhoid compared to not being vaccinated. For children aged 0–15 years, vaccination reduced the likelihood of testing positive by approximately 81.2% compared to those who were not vaccinated. Table 5 summarizes the effectiveness of TCV in Harare City.

**Table 5.** Vaccine Effectiveness (Direct) for Harare City, 2020–2024

Vaccination Status	Test Positive, n (%)	Test Negative, n (%)	Vaccine Effectiveness (95%)
**All ages**			
Not vaccinated	20 (15%)	113 (85%)	Ref
Vaccinated	4 (6%)	65 (94%)	61.4 (54.3–68.1)%
**Ages 0–15 y**			
Not vaccinated	18 (34%)	35 (66%)	Ref
Vaccinated	2 (6%)	30 (94%)	81.2 (71.2–88.8)%

### Comparative Analysis of *Salmonella* Typhi Genomes Before and After TCV

This study used genomic sequencing of 26 *Salmonella* Typhi isolates from confirmed typhoid cases to characterize the population structure and AMR profiles of the circulating strains before and after the TCV vaccination campaign in Harare.

Three isolates were excluded from further analysis, as 1 was identified as not being *Salmonella* Typhi, and the other 2 sequences were contaminated. The remaining isolates (n = 23) were analyzed along genomes from Harare isolated in previous studies [15, 16], representing a pool from before the vaccination campaign (n = 74).

**Figure 2.**
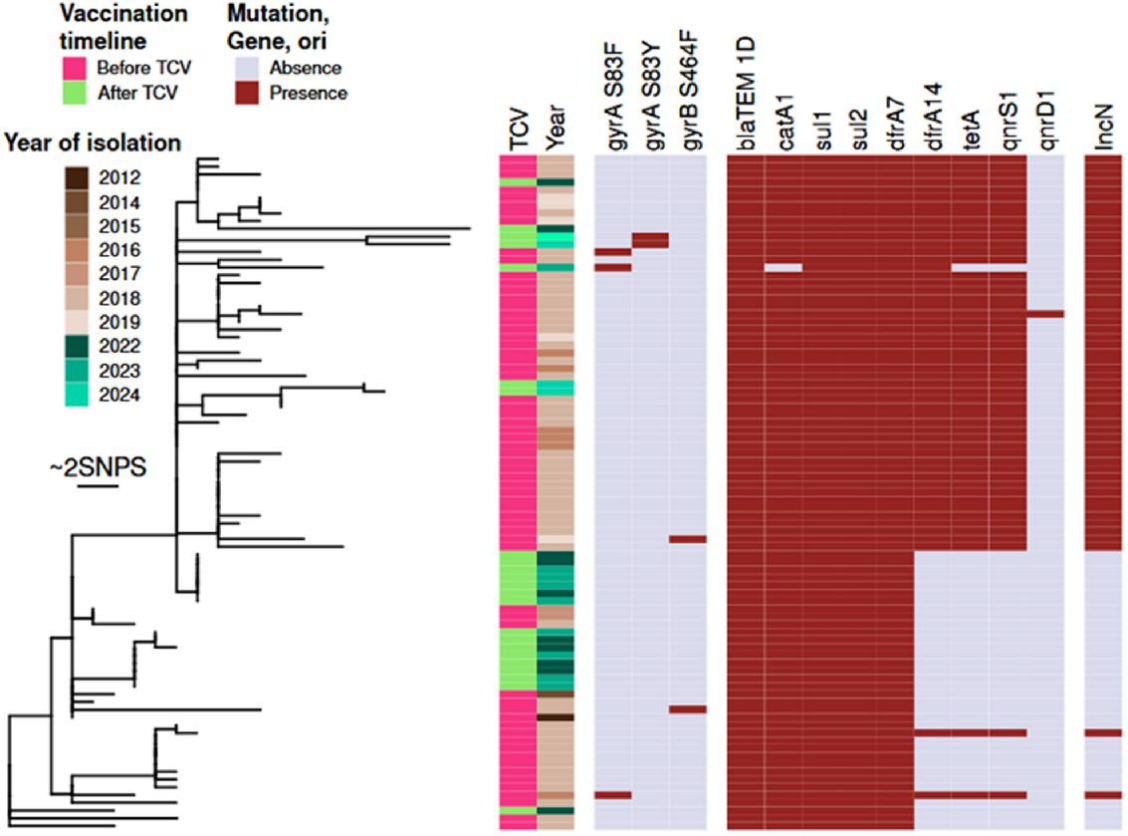
Phylogenetic tree and presence of ARG and ORI in *Salmonella* Typhi isolates.

All the newly sequenced isolates belong to the 4.3.1.1.EA1 genotype, like the majority (69 of 74) of the pre-TCV Harare isolates. A phylogenetic reconstruction of the 4.3.1.1.EA1 clade (Figure 2) shows that the post-TCV isolates are clustered despite being closely related to the pre-TCV population. All these pre- and post-TCV isolates had the β-lactamase *blaTEM-1D*, sulphonamide resistance genes *sul1* and *sul2*, and the trimethoprim resistance gene *dfrA7,* and all but 1 isolates carried the chloramphenicol resistance gene *catA1*.

There was no gene encoding resistance to additional class of antibiotics found in the post-TCV isolates, and their rate of resistance appeared lower overall. The frequency of strains containing mutations in *gyrA* and *gyrB,* that would be expected to confer resistance to fluoroquinolone antibiotics increased slightly following vaccination, with 5.8% (4/69) pre-TCV compared to 13% (3/23) post-TCV, although this difference was not statistically significant (Fisher exact test, *P* & .36). As previously reported the presence of an IncN origin of replication correlated with the presence of the *dfrA14*, *tetA,* and *qnrS1* encoding resistance to trimethoprim, tetracycline, and fluoroquinolone antibiotic, respectively [15, 16]. The combination of these genes and plasmid origin of replication was present in 66.7% (46/69) of the pre-TCV isolates, with an additional isolate carrying an IncN plasmid and *dfrA14* but not *tetA* nor *qnrS1*, and only in 30.4% (7/23) of the post-TCV isolates. The difference in frequency of isolates carrying the IncN plasmid with the 3 antibiotic resistance genes found before and after TCV was significant (Fisher exact test, *P* =. 003).

## DISCUSSION

The analysis considered typhoid trends, attack rates, and effectiveness of TCV in Harare City for the period 2017–2024. A total of 3401 cases were reported between 2017 and 2024, with the Western district, particularly Glen View and Budiriro, accounting for the highest number of cases. This is consistent with previous studies that have linked typhoid outbreaks in Harare to poor sanitation and unreliable water supply [11]. The age distribution of cases aligns with global trends, where children younger than age 16 years account for the majority of cases [11]. The high proportion of suspected cases reflects ongoing challenges in laboratory confirmation, a common issue in typhoid surveillance [15].

The introduction of TCV in 2019 achieved high coverage in targeted areas. However, the persistence of typhoid cases suggests that vaccination alone is insufficient, necessitating integrated, multisectoral interventions. There was a decline in attack rates across all districts of Harare City following the introduction of TCV. The Western district exhibited the most significant decline, followed by the Southern district. The finding is consistent with the results of a study in Karachi, Pakistan, which demonstrated a similar reduction in typhoid incidence following TCV introduction, with effectiveness rates exceeding 50% in controlled settings [17]. Moreover, previous outbreak investigations in Harare have linked high attack rates to contaminated water sources, suggesting that improved sanitation, plays a crucial role in disease control [18]. The Eastern district, which consistently had the lowest attack rates, may serve as a model for effective prevention strategies. These findings align with global trends in typhoid control, emphasizing the need for sustained immunization efforts [19].

The study findings show a decline in age-specific attack rates of *Salmonella* Typhi infections in Harare City following the introduction of TCV, particularly among younger age groups. Although reductions were observed across all age categories, none of the risk ratios reached statistical significance, indicating that factors other than vaccination may have influenced disease trends. Similar studies in Kenya have shown age-related variations in *Salmonella* seroprevalence, with younger children exhibiting higher susceptibility [20]. Additionally, global assessments of typhoid burden indicate that children aged 0–15 years bear the highest disease burden, reinforcing the importance of targeted immunization strategies [2, 5, 7].

Among age groups, the 0–15 age group demonstrated a higher vaccine effectiveness (81%) compared to the overall population (61.5%), suggesting greater benefit in younger populations. This aligns with evidence from a systematic review, which highlighted that children and adolescents demonstrate a robust immunogenic response to TCVs [21]. A field study conducted in Navi Mumbai, India, reported vaccine effectiveness of 80% among the 2- to 5-year age group, significantly reducing the risk of typhoid in the vaccinated populations [22]. This comparison highlights the potential of TCVs to reduce typhoid incidence substantially. Variability in vaccine effectiveness was observed across age groups in Harare mirrors findings from Bangladesh, where vaccine effectiveness ranged from 59% to 87% across different cohorts [23]. These disparities suggest that local factors, such as population density, WASH, and access to healthcare, may modulate vaccine impact.

The genome analysis revealed continued transmission of the same population of *Salmonella* Typhi isolates before and after the vaccination campaign. However post-TCV isolates were less diverse with the majority clustering together in the population structure. This may be the result of sample bias but may also indicate that the TCV program was more effective in some geographical locations of Harare than others.

Previous reports indicate that distinct genotypes are present in geographical locations. These genomic observations are consistent with different declines in attack rates in areas within Harare. A larger genomic study is required to investigate if the genotypes that dominate post-TCV are specific to regions where the attack rate was not significantly reduced. A smaller proportion of the post-TCV population carried the IncN plasmid compared to the pre-TCV population. This results in a lower rate of isolates carrying the *qnrS* gene. These findings are in line with phenotypic resistance data, which also indicate reduced resistance to ciprofloxacin. Whether this change is due to sampling bias or it can be linked to the vaccination campaign should be investigated in the future.

The deployment of TCVs represents a critical intervention in the global effort to combat multidrug-resistant typhoid. TCVs have demonstrated high efficacy in reducing the incidence of typhoid fever, thereby decreasing the overall disease burden [24]. Decreased transmission leads to decreased need for antibiotics, the overuse of which leads to antibiotic resistance. Moreover, widespread immunization helps build herd immunity, offering indirect protection to unvaccinated individuals and contributing to community-level disease control.

From a health economics perspective, several studies have demonstrated that TCVs are cost-effective in endemic regions, particularly when considering the high costs of managing multidrug-resistant and XDR typhoid infections [25, 26]. However, it is equally important to recognize that investments in WASH is also highly cost-effective, with benefits extending beyond typhoid to other enteric diseases. Thus, vaccination should be viewed as a complementary strategy to WASH improvements: TCVs provide immediate protection and reduce the burden of resistant infections, whereas WASH investments address the underlying drivers of transmission and offer sustainable long-term health gains. Our genomic analysis did not demonstrate marked differences post-TCV, underscoring the need to interpret reductions in incidence within the broader context of concurrent WASH interventions.

Although the association between TCV introduction and reduced typhoid incidence in Harare is notable, multisectoral interventions, particularly improvements in WASH, could have also contributed significantly to disease control. Modeling studies in Harare demonstrated that WASH interventions, including improved water supply and sanitation infrastructure, were critical in reducing transmission during outbreaks [27]. Organizations such as Médecins Sans Frontières and UNICEF implemented targeted WASH programs, providing safe water access and hygiene promotion in affected communities. These efforts, in addition to vaccination, could have created a synergistic effect that likely accelerated the decline in typhoid cases.

This study had some limitations. Missing data for 2020 and 2021 because of the COVID-19 pandemic may have contributed to underestimation of disease burden. Reduced case numbers could partly reflect gaps in reporting rather than true declines. The lack of detailed data on WASH and other contributing factors prevented a comprehensive assessment of their role in transmission. Although TCV introduction was associated with reduced typhoid incidence, concurrent WASH improvements could have contributed substantially to disease control. Finally, the generalizability of findings may be limited to urban settings with similar epidemiological characteristics, necessitating further research in rural areas and diverse contexts.

In conclusion, the TCV campaign in Harare City significantly lowered typhoid attack rates, especially in high-incidence areas like the Western suburbs, where cases dropped notably. Younger age groups benefited from higher vaccine effectiveness, indicating a need to focus on these populations in vaccination efforts. TCVs offer a scientifically validated and economically viable strategy for controlling multidrug-resistant *Salmonella* Typhi by significantly reducing infection rates [24], curbing antibiotic usage, enhancing herd immunity, and mitigating the public health impact of antimicrobial resistance. To achieve sustained control and eventual elimination of typhoid fever, vaccination efforts must be complemented by investments in WASH infrastructure, enhanced disease surveillance, and community health education. These findings support global evidence for combined public health approaches and stress the importance of ongoing monitoring and research. Future research should explore factors affecting vaccine performance across different age groups and evaluate the cost-effectiveness of strategies that combine vaccination and WASH improvements. The findings provide invaluable evidence to inform policy decisions and support the scaling up of TCV.

## Supplementary Material

ofag091_Supplementary_Data

## Supplementary Data

Supplementary materials are available at *Open Forum Infectious Diseases* online. Consisting of data provided by the authors to benefit the reader, the posted materials are not copyedited and are the sole responsibility of the authors, so questions or comments should be addressed to the corresponding author.

## Notes

***Author Contributions*.** T.B., J.M., G.T., M.V., I.M., D.N., F.C., A.M., A.J., P.C., I.P., A.S., B.C., M.M., W.F., P.S., P.N., R.K., R.L., G.M., and T.M. were involved in the conception and design of the study. T.B., M.M., J.M., and M.V. performed the data collation and analysis. T.B., J.M., M.M., T.M., I.M., and M.V. accessed and verified all the data. T.B., T.M., M.V., J.M., D.N., F.C., I.M., and M.V. wrote the first draft. T.B., T.M., J.M., G.M., A.M., R.L., A.J., R.K., G.T., P.N., I.M., P.S., D.N., W.F., F.C., M.M., M.V., B.C., P.C., A.S., I.P., and B.V. interpreted the data and provided intellectual input, including editing and reviewing the final draft. All authors had full access to all the data in the study and had final responsibility for the decision to submit for publication.

***Data sharing*.** Deidentified data that include key variables described in the peer-reviewed publication will be made available upon a reasonable request after the publication of the peer-reviewed article.

***Financial support.*** Funding for this study was provided by the AMR Multi-Partner Trust Fund (AMR MPTF 00127114), and UK Fleming Fund, (FF167569).

***Potential conflicts of interest*.** The authors: No reported conflicts of interest. All authors have submitted the ICMJE Form for Disclosure of Potential Conflicts of Interest.
