# Supplementary material for: From Crisis to Control: A Study of Typhoid Conjugate Vaccine Efficacy in Harare, Zimbabwe (2017–2024)
Source: Open Forum Infect Dis. 2026 Mar 16;13(3):ofag091. doi: 10.1093/ofid/ofag091 (PMC13006139; doi:10.1093/ofid/ofag091)
Supplement: ofag091_Supplementary_Data [file ofag091_supplementary_data.docx]

**From Crisis to Control: A Study of Typhoid Conjugate Vaccine Efficacy in Harare, Zimbabwe (2017–2024)**

**Authors:**

Talent Bvochora, M.D., M.P.H.^1^, John Manyara, M.P.H^1^, Augustine Muzondo, MSc^2^, Agnes Juru, MPH^2^, Gaetan Thilliez, Ph.D^3^, Innocent Mukeredzi, Ph.D^1^, Denford Nhamo^1^, Farai Chitiyo^4^, Michael Vere, M.D., M.P.H.^1^, Prosper Chonzi, M.D., M.P.H.^1^, Isaac Phiri, M.D., M.P.H^5^, Anthony M. Smith, PhD^6,7^, Blessmore V Chaibva^8^, Munyaradzi Mapingure, MSc^9^, Walter Fuller, MD., MBA^10^, Pramila Shrestha, MSc^11^, Parvati Nair, M.B.B.S., DTMH, MTM^12^, Robert A. Kingsley, PhD^13,14^, Ramanan Laxminarayan, PhD ^15,16^, Godfrey Musuka, D.V.M, Ph.D^17^, Tapfumanei Mashe, Ph.D, MTM^18^

**Affiliations:**

1. Harare City Health Department, Harare, Zimbabwe
2. National Microbiology Reference laboratory, Harare, Zimbabwe
3. School of Biotechnology, Dublin City University, Dublin, Ireland
4. Harare Metropolitan Province, Medical Directorate, Harare, Zimbabwe
5. Epidemiology and Disease Control, Ministry of Health and Child Care, Harare, Zimbabwe
6. Centre for Enteric Diseases, National Institute for Communicable Diseases, Division of the National Health Laboratory Service, Johannesburg, South Africa
7. Department of Medical Microbiology, Faculty of Health Sciences, University of Pretoria, Pretoria, South Africa
8. Department of Pharmacy , Ministry of Health and Child Care, Harare, Zimbabwe
9. Department of Global Public Health and Family Medicine, Faculty of Medicine and Health Sciences, University of Zimbabwe, Harare, Zimbabwe
10. WHO Regional Office for Africa (AFRO), Cité du Djoué, P.O. Box 06, Brazzaville, Republic of Congo
11. World Health Organization, Geneva, Switzerland
12. Médecins Sans Frontières (MSF) South Asia, Colombo, Sri Lanka
13. Quadram Institute Bioscience, Norwich, United Kingdom
14. University of East Anglia, Norwich, UK
15. One Health Trust, Bengaluru, India
16. High Meadows Environmental Institute, Princeton University, Princeton, NJ, USA
17. 3ieimpact, Harare, Zimbabwe
18. Health Systems Strengthening Unit, World Health Organization, Harare, Zimbabwe

**Corresponding authors:** Talent Bvochora, [tbvochora@yahoo.com](mailto:tbvochora@yahoo.com) and Tapfumanei Mashe, [mashet2006@yahoo.co.uk](mailto:mashet2006@yahoo.co.uk)

**Table of Contents**

Genome Analysis 3

References 3

Supplementary Table 1. Accession and genomic information 7

**Supplementary methods:**

# **Genome analysis**

Read QC was carried out using Fastp v0.23.4 (1) and MultiQC v1.22.2 (2). Mykrobe was ran using the typhi module to get in silico confirmation of the serotypes, genotyphi genotypes (3) , the presence of antibiotic resistance genes and mutations, and presence of plasmids origin of replication. Genomes were assembled using shovill v1.1.0 with the SPAdes assembler (4) and the quality of the assembled genome was assessed using checkm2 (5). All sequences with less than 141MB (theoretical coverage of 30X for a 4.7mb genome) in the MultiQC report, identified as not being *S.* Typhi by Mykrobe or detected as contaminated with checkm2 were excluded.

Core alignment against reference CT18 (NC003198.1) was carried out using snippy v4.6.0 (6) . Genomes were analysed along with isolates from previous studies (7,8). Gubbins (9) was used to detect recombination region and mask them from the full alignment, then core-snp-filter (10) generate an alignment with SNP found in at least 95% of the isolates. This alignment was used to build a phylogenetic tree using RAxML-NG (11) with the GTRGAMMA model, default tree building options, and a 1000 bootstrap with the autoMRE option enabled. Tree plotted in Rstudio (12) using ggtree (13) and ape (14).

**References:**

1. Chen S. Ultrafast one-pass FASTQ data preprocessing, quality control, and deduplication using fastp. iMeta. 2023;2(2):e107.

2. Ewels P, Magnusson M, Lundin S, Käller M. MultiQC: summarize analysis results for multiple tools and samples in a single report. Bioinformatics. 2016 Oct 1;32(19):3047–8.

3. Dyson ZA, Holt KE. Five Years of GenoTyphi: Updates to the Global Salmonella Typhi Genotyping Framework. J Infect Dis. 2021 Dec 15;224(Supplement_7):S775–80.

4. Prjibelski A, Antipov D, Meleshko D, Lapidus A, Korobeynikov A. Using SPAdes De Novo Assembler. Curr Protoc Bioinforma. 2020;70(1):e102.

5. CheckM2: a rapid, scalable and accurate tool for assessing microbial genome quality using machine learning | Nature Methods [Internet]. [cited 2025 Jul 7]. Available from: https://www.nature.com/articles/s41592-023-01940-w

6. Seemann T. tseemann/shovill [Internet]. 2025 [cited 2025 Jul 7]. Available from: https://github.com/tseemann/shovill

7. Mashe T, Leekitcharoenphon P, Mtapuri-Zinyowera S, Kingsley RA, Robertson V, Tarupiwa A, et al. Salmonella enterica serovar Typhi H58 clone has been endemic in Zimbabwe from 2012 to 2019. J Antimicrob Chemother. 2021 May 1;76(5):1160–7.

8. Thilliez G, Mashe T, Chaibva BV, Robertson V, Bawn M, Tarupiwa A, et al. Population structure of Salmonella enterica Typhi in Harare, Zimbabwe (2012–19) before typhoid conjugate vaccine roll-out: a genomic epidemiology study. Lancet Microbe. 2023 Dec 1;4(12):e1005–14.

9. Croucher NJ, Page AJ, Connor TR, Delaney AJ, Keane JA, Bentley SD, et al. Rapid phylogenetic analysis of large samples of recombinant bacterial whole genome sequences using Gubbins. Nucleic Acids Res. 2015 Feb 18;43(3):e15.

10. Taouk ML, Featherstone LA, Taiaroa G, Seemann T, Ingle DJ, Stinear TP, et al. Exploring SNP filtering strategies: the influence of strict vs soft core. Microb Genomics. 2025;11(1):001346.

11. Kozlov AM, Darriba D, Flouri T, Morel B, Stamatakis A. RAxML-NG: a fast, scalable and user-friendly tool for maximum likelihood phylogenetic inference. Bioinformatics. 2019 Nov 1;35(21):4453–5.

12. Posit [Internet]. [cited 2025 Jul 7]. Posit. Available from: https://www.posit.co/

13. Yu G, Smith DK, Zhu H, Guan Y, Lam TTY. ggtree: an r package for visualization and annotation of phylogenetic trees with their covariates and other associated data. Methods Ecol Evol. 2017;8(1):28–36.

14. Paradis E, Schliep K. ape 5.0: an environment for modern phylogenetics and evolutionary analyses in R. Bioinformatics. 2019 Feb 1;35(3):526–8.

**Supplementary Table 1 . Accession and genomic information**

| Read accession | BioProject | BioSample | sample_name | Year | Town | study | genotype | IncN | gyrA S83F | gyrA S83Y | gyrB S464F | blaTEM-1D | qnrS1 | qnrD1 | catA1 | sul1 | sul2 | dfrA7 | dfrA14 | tetA |
| --- | --- | --- | --- | --- | --- | --- | --- | --- | --- | --- | --- | --- | --- | --- | --- | --- | --- | --- | --- | --- |
| Pending assignment | PRJNA1309314 | SAMN50744236 | NMRL22120603 | 2022 | Harare | This study | 4.3.1.1.EA1 | 0 | 0 | 0 | 0 | 1 | 0 | 0 | 1 | 1 | 1 | 1 | 0 | 0 |
| Pending assignment | PRJNA1309314 | SAMN50744237 | NMRL22120610 | 2022 | Harare | This study | 4.3.1.1.EA1 | 0 | 0 | 0 | 0 | 1 | 0 | 0 | 1 | 1 | 1 | 1 | 0 | 0 |
| Pending assignment | PRJNA1309314 | SAMN50744238 | NMRL22120614 | 2022 | Harare | This study | 4.3.1.1.EA1 | 0 | 0 | 0 | 0 | 1 | 0 | 0 | 1 | 1 | 1 | 1 | 0 | 0 |
| Pending assignment | PRJNA1309314 | SAMN50744239 | NMRL22120615 | 2022 | Harare | This study | 4.3.1.1.EA1 | 1 | 0 | 0 | 0 | 1 | 1 | 0 | 1 | 1 | 1 | 1 | 1 | 1 |
| Pending assignment | PRJNA1309314 | SAMN50744240 | NMRL22120616 | 2022 | Harare | This study | 4.3.1.1.EA1 | 0 | 0 | 0 | 0 | 1 | 0 | 0 | 1 | 1 | 1 | 1 | 0 | 0 |
| Pending assignment | PRJNA1309314 | SAMN50744241 | NMRL22120633 | 2022 | Harare | This study | 4.3.1.1.EA1 | 0 | 0 | 0 | 0 | 1 | 0 | 0 | 1 | 1 | 1 | 1 | 0 | 0 |
| Pending assignment | PRJNA1309314 | SAMN50744242 | NMRL23010015 | 2023 | Harare | This study | 4.3.1.1.EA1 | 0 | 0 | 0 | 0 | 1 | 0 | 0 | 1 | 1 | 1 | 1 | 0 | 0 |
| Pending assignment | PRJNA1309314 | SAMN50744243 | NMRL23010016 | 2023 | Harare | This study | 4.3.1.1.EA1 | 0 | 0 | 0 | 0 | 1 | 0 | 0 | 1 | 1 | 1 | 1 | 0 | 0 |
| Pending assignment | PRJNA1309314 | SAMN50744244 | NMRL23010017 | 2023 | Harare | This study | 4.3.1.1.EA1 | 1 | 1 | 0 | 0 | 1 | 0 | 0 | 0 | 1 | 1 | 1 | 1 | 0 |
| Pending assignment | PRJNA1309314 | SAMN50744245 | NMRL23010019 | 2023 | Harare | This study | 4.3.1.1.EA1 | 0 | 0 | 0 | 0 | 1 | 0 | 0 | 1 | 1 | 1 | 1 | 0 | 0 |
| Pending assignment | PRJNA1309314 | SAMN50744246 | NMRL23010020 | 2023 | Harare | This study | 4.3.1.1.EA1 | 0 | 0 | 0 | 0 | 1 | 0 | 0 | 1 | 1 | 1 | 1 | 0 | 0 |
| Pending assignment | PRJNA1309314 | SAMN50744247 | NMRL23010080 | 2023 | Harare | This study | 4.3.1.1.EA1 | 1 | 0 | 0 | 0 | 1 | 1 | 0 | 1 | 1 | 1 | 1 | 1 | 1 |
| Pending assignment | PRJNA1309314 | SAMN50744248 | NMRL23030081 | 2023 | Harare | This study | 4.3.1.1.EA1 | 0 | 0 | 0 | 0 | 1 | 0 | 0 | 1 | 1 | 1 | 1 | 0 | 0 |
| Pending assignment | PRJNA1309314 | SAMN50744249 | NMRL23121435 | 2023 | Harare | This study | 4.3.1.1.EA1 | 0 | 0 | 0 | 0 | 1 | 0 | 0 | 1 | 1 | 1 | 1 | 0 | 0 |
| Pending assignment | PRJNA1309314 | SAMN50744250 | NMRL23121436 | 2023 | Harare | This study | 4.3.1.1.EA1 | 0 | 0 | 0 | 0 | 1 | 0 | 0 | 1 | 1 | 1 | 1 | 0 | 0 |
| Pending assignment | PRJNA1309314 | SAMN50744251 | NMRL23121437 | 2023 | Harare | This study | 4.3.1.1.EA1 | 0 | 0 | 0 | 0 | 1 | 0 | 0 | 1 | 1 | 1 | 1 | 0 | 0 |
| Pending assignment | PRJNA1309314 | SAMN50744252 | NMRL23121439 | 2023 | Harare | This study | 4.3.1.1.EA1 | 0 | 0 | 0 | 0 | 1 | 0 | 0 | 1 | 1 | 1 | 1 | 0 | 0 |
| Pending assignment | PRJNA1309314 | SAMN50744253 | NMRL24010060 | 2024 | Harare | This study | 4.3.1.1.EA1 | 1 | 0 | 0 | 0 | 1 | 1 | 0 | 1 | 1 | 1 | 1 | 1 | 1 |
| Pending assignment | PRJNA1309314 | SAMN50744254 | NMRL24010061 | 2024 | Harare | This study | 4.3.1.1.EA1 | 1 | 0 | 0 | 0 | 1 | 1 | 0 | 1 | 1 | 1 | 1 | 1 | 1 |
| Pending assignment | PRJNA1309314 | SAMN50744255 | NMRL24040425 | 2024 | Harare | This study | 4.3.1.1.EA1 | 1 | 0 | 1 | 0 | 1 | 1 | 0 | 1 | 1 | 1 | 1 | 1 | 1 |
| Pending assignment | PRJNA1309314 | SAMN50744256 | NMRL24040426 | 2024 | Harare | This study | 4.3.1.1.EA1 | 1 | 0 | 1 | 0 | 1 | 1 | 0 | 1 | 1 | 1 | 1 | 1 | 1 |
| ERR4870975 | PRJEB41494 | SAMEA7616098 | HG3-1 | 2012 | Harare | Mashe et al 2021 https://doi.org/10.1093/jac/dkaa519 | 4.3.1.1.EA1 | 0 | 0 | 0 | 0 | 1 | 0 | 0 | 1 | 1 | 1 | 1 | 0 | 0 |
| ERR4870976 | PRJEB41494 | SAMEA7616099 | HB1-2 | 2014 | Harare | Mashe et al 2021 https://doi.org/10.1093/jac/dkaa519 | 4.3.1.1.EA1 | 0 | 0 | 0 | 0 | 1 | 0 | 0 | 1 | 1 | 1 | 1 | 0 | 0 |
| ERR4870977 | PRJEB41494 | SAMEA7616100 | HG8-3 | 2016 | Harare | Mashe et al 2021 https://doi.org/10.1093/jac/dkaa519 | 4.3.1.1.EA1 | 1 | 0 | 0 | 0 | 1 | 1 | 0 | 1 | 1 | 1 | 1 | 1 | 1 |
| ERR4870978 | PRJEB41494 | SAMEA7616101 | HK3-4 | 2016 | Harare | Mashe et al 2021 https://doi.org/10.1093/jac/dkaa519 | 4.3.1.1.EA1 | 1 | 0 | 0 | 0 | 1 | 1 | 0 | 1 | 1 | 1 | 1 | 1 | 1 |
| ERR4870979 | PRJEB41494 | SAMEA7616102 | HB1-5 | 2016 | Harare | Mashe et al 2021 https://doi.org/10.1093/jac/dkaa519 | 4.3.1.1.EA1 | 1 | 0 | 0 | 0 | 1 | 1 | 0 | 1 | 1 | 1 | 1 | 1 | 1 |
| ERR4870980 | PRJEB41494 | SAMEA7616103 | HB5-6 | 2016 | Harare | Mashe et al 2021 https://doi.org/10.1093/jac/dkaa519 | 4.3.1.1.EA1 | 1 | 0 | 0 | 0 | 1 | 1 | 0 | 1 | 1 | 1 | 1 | 1 | 1 |
| ERR4870981 | PRJEB41494 | SAMEA7616104 | HB1-7 | 2016 | Harare | Mashe et al 2021 https://doi.org/10.1093/jac/dkaa519 | 4.3.1.1.EA1 | 1 | 0 | 0 | 0 | 1 | 1 | 0 | 1 | 1 | 1 | 1 | 1 | 1 |
| ERR4870982 | PRJEB41494 | SAMEA7616105 | HM-8 | 2016 | Harare | Mashe et al 2021 https://doi.org/10.1093/jac/dkaa519 | 4.3.1.1.EA1 | 1 | 1 | 0 | 0 | 1 | 1 | 0 | 1 | 1 | 1 | 1 | 1 | 1 |
| ERR4870983 | PRJEB41494 | SAMEA7616106 | HM-11 | 2017 | Harare | Mashe et al 2021 https://doi.org/10.1093/jac/dkaa519 | 3.3.1 | 0 | 0 | 0 | 0 | 0 | 0 | 0 | 0 | 0 | 0 | 0 | 0 | 0 |
| ERR4870984 | PRJEB41494 | SAMEA7616107 | HD-9 | 2017 | Harare | Mashe et al 2021 https://doi.org/10.1093/jac/dkaa519 | 4.3.1.1.EA1 | 0 | 0 | 0 | 0 | 1 | 0 | 0 | 1 | 1 | 1 | 1 | 0 | 0 |
| ERR4870985 | PRJEB41494 | SAMEA7616108 | HM-9 | 2017 | Harare | Mashe et al 2021 https://doi.org/10.1093/jac/dkaa519 | 3.3.1 | 0 | 0 | 0 | 0 | 0 | 0 | 0 | 0 | 0 | 0 | 0 | 0 | 0 |
| ERR4870986 | PRJEB41494 | SAMEA7616109 | HD-10 | 2017 | Harare | Mashe et al 2021 https://doi.org/10.1093/jac/dkaa519 | 4.3.1.1.EA1 | 0 | 0 | 0 | 0 | 1 | 0 | 0 | 1 | 1 | 1 | 1 | 0 | 0 |
| ERR4870987 | PRJEB41494 | SAMEA7616110 | HG3-15 | 2018 | Harare | Mashe et al 2021 https://doi.org/10.1093/jac/dkaa519 | 4.3.1.1.EA1 | 1 | 0 | 0 | 0 | 1 | 1 | 0 | 1 | 1 | 1 | 1 | 1 | 1 |
| ERR4870988 | PRJEB41494 | SAMEA7616111 | HG1-14 | 2018 | Harare | Mashe et al 2021 https://doi.org/10.1093/jac/dkaa519 | 4.3.1.1.EA1 | 1 | 0 | 0 | 0 | 1 | 1 | 0 | 1 | 1 | 1 | 1 | 1 | 1 |
| ERR4870989 | PRJEB41494 | SAMEA7616112 | HG3-13 | 2018 | Harare | Mashe et al 2021 https://doi.org/10.1093/jac/dkaa519 | 4.3.1.1.EA1 | 1 | 0 | 0 | 0 | 1 | 1 | 0 | 1 | 1 | 1 | 1 | 1 | 1 |
| ERR4870990 | PRJEB41494 | SAMEA7616113 | HK3-16 | 2018 | Harare | Mashe et al 2021 https://doi.org/10.1093/jac/dkaa519 | 4.3.1.1.EA1 | 1 | 0 | 0 | 0 | 1 | 1 | 0 | 1 | 1 | 1 | 1 | 1 | 1 |
| ERR4870991 | PRJEB41494 | SAMEA7616114 | HS-17 | 2018 | Harare | Mashe et al 2021 https://doi.org/10.1093/jac/dkaa519 | 4.3.1.1.EA1 | 1 | 0 | 0 | 0 | 1 | 1 | 0 | 1 | 1 | 1 | 1 | 1 | 1 |
| ERR4870992 | PRJEB41494 | SAMEA7616115 | HK3-18 | 2018 | Harare | Mashe et al 2021 https://doi.org/10.1093/jac/dkaa519 | 4.3.1.1.EA1 | 1 | 0 | 0 | 0 | 1 | 1 | 0 | 1 | 1 | 1 | 1 | 1 | 1 |
| ERR4870993 | PRJEB41494 | SAMEA7616116 | HH-19 | 2018 | Harare | Mashe et al 2021 https://doi.org/10.1093/jac/dkaa519 | 4.3.1.1.EA1 | 1 | 0 | 0 | 0 | 1 | 1 | 0 | 1 | 1 | 1 | 1 | 1 | 1 |
| ERR4870999 | PRJEB41494 | SAMEA7616122 | HM-25 | 2019 | Harare | Mashe et al 2021 https://doi.org/10.1093/jac/dkaa519 | 4.3.1.1.EA1 | 1 | 0 | 0 | 1 | 1 | 1 | 0 | 1 | 1 | 1 | 1 | 1 | 1 |
| ERR4871000 | PRJEB41494 | SAMEA7616123 | HB-26 | 2019 | Harare | Mashe et al 2021 https://doi.org/10.1093/jac/dkaa519 | 4.3.1.1.EA1 | 1 | 0 | 0 | 0 | 1 | 1 | 0 | 1 | 1 | 1 | 1 | 1 | 1 |
| ERR4871001 | PRJEB41494 | SAMEA7616124 | HB-27 | 2019 | Harare | Mashe et al 2021 https://doi.org/10.1093/jac/dkaa519 | 4.3.1.1.EA1 | 1 | 0 | 0 | 0 | 1 | 1 | 0 | 1 | 1 | 1 | 1 | 1 | 1 |
| ERR4871002 | PRJEB41494 | SAMEA7616125 | HB-28 | 2019 | Harare | Mashe et al 2021 https://doi.org/10.1093/jac/dkaa519 | 4.3.1.1.EA1 | 1 | 0 | 0 | 0 | 1 | 1 | 0 | 1 | 1 | 1 | 1 | 1 | 1 |
| ERR4871003 | PRJEB41494 | SAMEA7616126 | HB-29 | 2019 | Harare | Mashe et al 2021 https://doi.org/10.1093/jac/dkaa519 | 4.3.1.1.EA1 | 1 | 0 | 0 | 0 | 1 | 1 | 0 | 1 | 1 | 1 | 1 | 1 | 1 |
| SRR20666348 | PRJNA862761 | SAMN29993554 | S_Typhi_QIB-NM118 | 2018 | Harare | Thilliez et al 2023 http://doi.org/10.1016/s2666-5247(23)00214-8 | 4.3.1.1.EA1 | 1 | 0 | 0 | 0 | 1 | 1 | 0 | 1 | 1 | 1 | 1 | 1 | 1 |
| SRR20666349 | PRJNA862761 | SAMN29993553 | S_Typhi_QIB-NM116 | 2018 | Harare | Thilliez et al 2023 http://doi.org/10.1016/s2666-5247(23)00214-8 | 4.3.1.1.EA1 | 1 | 0 | 0 | 0 | 1 | 1 | 0 | 1 | 1 | 1 | 1 | 1 | 1 |
| SRR20666350 | PRJNA862761 | SAMN29993552 | S_Typhi_QIB-NM115 | 2018 | Harare | Thilliez et al 2023 http://doi.org/10.1016/s2666-5247(23)00214-8 | 4.3.1.1.EA1 | 0 | 0 | 0 | 0 | 1 | 0 | 0 | 1 | 1 | 1 | 1 | 0 | 0 |
| SRR20666351 | PRJNA862761 | SAMN29993602 | S_Typhi_QIB-NM151 | 2018 | Harare | Thilliez et al 2023 http://doi.org/10.1016/s2666-5247(23)00214-8 | 3.3.1 | 0 | 0 | 0 | 0 | 0 | 0 | 0 | 0 | 0 | 0 | 0 | 0 | 0 |
| SRR20666352 | PRJNA862761 | SAMN29993601 | S_Typhi_QIB-NM141 | 2018 | Harare | Thilliez et al 2023 http://doi.org/10.1016/s2666-5247(23)00214-8 | 3.3.1 | 0 | 0 | 0 | 0 | 0 | 0 | 0 | 0 | 0 | 0 | 0 | 0 | 0 |
| SRR20666353 | PRJNA862761 | SAMN29993600 | S_Typhi_QIB-NM131 | 2018 | Harare | Thilliez et al 2023 http://doi.org/10.1016/s2666-5247(23)00214-8 | 3.3.1 | 0 | 0 | 0 | 0 | 0 | 0 | 0 | 0 | 0 | 0 | 0 | 0 | 0 |
| SRR20666354 | PRJNA862761 | SAMN29993599 | S_Typhi_QIB-NM86 | 2018 | Harare | Thilliez et al 2023 http://doi.org/10.1016/s2666-5247(23)00214-8 | 4.3.1.1.EA1 | 1 | 0 | 0 | 0 | 1 | 1 | 0 | 1 | 1 | 1 | 1 | 1 | 1 |
| SRR20666355 | PRJNA862761 | SAMN29993598 | S_Typhi_QIB-NM165 | 2018 | Harare | Thilliez et al 2023 http://doi.org/10.1016/s2666-5247(23)00214-8 | 4.3.1.1.EA1 | 1 | 0 | 0 | 0 | 1 | 1 | 0 | 1 | 1 | 1 | 1 | 1 | 1 |
| SRR20666357 | PRJNA862761 | SAMN29993596 | S_Typhi_QIB-NM163 | 2018 | Harare | Thilliez et al 2023 http://doi.org/10.1016/s2666-5247(23)00214-8 | 4.3.1.1.EA1 | 1 | 0 | 0 | 0 | 1 | 1 | 0 | 1 | 1 | 1 | 1 | 1 | 1 |
| SRR20666358 | PRJNA862761 | SAMN29993551 | S_Typhi_QIB-NM114 | 2018 | Harare | Thilliez et al 2023 http://doi.org/10.1016/s2666-5247(23)00214-8 | 4.3.1.1.EA1 | 0 | 0 | 0 | 0 | 1 | 0 | 0 | 1 | 1 | 1 | 1 | 0 | 0 |
| SRR20666360 | PRJNA862761 | SAMN29993594 | S_Typhi_QIB-NM161 | 2018 | Harare | Thilliez et al 2023 http://doi.org/10.1016/s2666-5247(23)00214-8 | 4.3.1.1.EA1 | 1 | 0 | 0 | 0 | 1 | 1 | 0 | 1 | 1 | 1 | 1 | 1 | 1 |
| SRR20666361 | PRJNA862761 | SAMN29993593 | S_Typhi_QIB-NM160 | 2018 | Harare | Thilliez et al 2023 http://doi.org/10.1016/s2666-5247(23)00214-8 | 4.3.1.1.EA1 | 0 | 0 | 0 | 0 | 1 | 0 | 0 | 1 | 1 | 1 | 1 | 0 | 0 |
| SRR20666362 | PRJNA862761 | SAMN29993592 | S_Typhi_QIB-NM159 | 2018 | Harare | Thilliez et al 2023 http://doi.org/10.1016/s2666-5247(23)00214-8 | 4.3.1.1.EA1 | 0 | 0 | 0 | 0 | 1 | 0 | 0 | 1 | 1 | 1 | 1 | 0 | 0 |
| SRR20666363 | PRJNA862761 | SAMN29993591 | S_Typhi_QIB-NM158 | 2018 | Harare | Thilliez et al 2023 http://doi.org/10.1016/s2666-5247(23)00214-8 | 4.3.1.1.EA1 | 1 | 0 | 0 | 0 | 1 | 1 | 0 | 1 | 1 | 1 | 1 | 1 | 1 |
| SRR20666364 | PRJNA862761 | SAMN29993590 | S_Typhi_QIB-NM157 | 2018 | Harare | Thilliez et al 2023 http://doi.org/10.1016/s2666-5247(23)00214-8 | 4.3.1.1.EA1 | 0 | 0 | 0 | 0 | 1 | 0 | 0 | 1 | 1 | 1 | 1 | 0 | 0 |
| SRR20666366 | PRJNA862761 | SAMN29993588 | S_Typhi_QIB-NM155 | 2018 | Harare | Thilliez et al 2023 http://doi.org/10.1016/s2666-5247(23)00214-8 | 4.3.1.1.EA1 | 1 | 0 | 0 | 0 | 1 | 1 | 0 | 1 | 1 | 1 | 1 | 1 | 1 |
| SRR20666367 | PRJNA862761 | SAMN29993587 | S_Typhi_QIB-NM154 | 2018 | Harare | Thilliez et al 2023 http://doi.org/10.1016/s2666-5247(23)00214-8 | 4.3.1.1.EA1 | 1 | 0 | 0 | 0 | 1 | 1 | 0 | 1 | 1 | 1 | 1 | 1 | 1 |
| SRR20666369 | PRJNA862761 | SAMN29993550 | S_Typhi_QIB-NM113 | 2018 | Harare | Thilliez et al 2023 http://doi.org/10.1016/s2666-5247(23)00214-8 | 4.3.1.1.EA1 | 1 | 1 | 0 | 0 | 1 | 1 | 0 | 1 | 1 | 1 | 1 | 1 | 1 |
| SRR20666370 | PRJNA862761 | SAMN29993585 | S_Typhi_QIB-NM149 | 2018 | Harare | Thilliez et al 2023 http://doi.org/10.1016/s2666-5247(23)00214-8 | 4.3.1.1.EA1 | 1 | 0 | 0 | 0 | 1 | 1 | 0 | 1 | 1 | 1 | 1 | 1 | 1 |
| SRR20666371 | PRJNA862761 | SAMN29993584 | S_Typhi_QIB-NM148 | 2018 | Harare | Thilliez et al 2023 http://doi.org/10.1016/s2666-5247(23)00214-8 | 4.3.1.1.EA1 | 1 | 0 | 0 | 0 | 1 | 1 | 0 | 1 | 1 | 1 | 1 | 1 | 1 |
| SRR20666372 | PRJNA862761 | SAMN29993583 | S_Typhi_QIB-NM147 | 2018 | Harare | Thilliez et al 2023 http://doi.org/10.1016/s2666-5247(23)00214-8 | 4.3.1.1.EA1 | 1 | 0 | 0 | 0 | 1 | 1 | 0 | 1 | 1 | 1 | 1 | 1 | 1 |
| SRR20666373 | PRJNA862761 | SAMN29993582 | S_Typhi_QIB-NM145 | 2018 | Harare | Thilliez et al 2023 http://doi.org/10.1016/s2666-5247(23)00214-8 | 4.3.1.1.EA1 | 0 | 0 | 0 | 0 | 1 | 0 | 0 | 1 | 1 | 1 | 1 | 0 | 0 |
| SRR20666374 | PRJNA862761 | SAMN29993581 | S_Typhi_QIB-NM144 | 2018 | Harare | Thilliez et al 2023 http://doi.org/10.1016/s2666-5247(23)00214-8 | 4.3.1.1.EA1 | 1 | 0 | 0 | 0 | 1 | 1 | 0 | 1 | 1 | 1 | 1 | 1 | 1 |
| SRR20666375 | PRJNA862761 | SAMN29993580 | S_Typhi_QIB-NM143 | 2018 | Harare | Thilliez et al 2023 http://doi.org/10.1016/s2666-5247(23)00214-8 | 4.3.1.1.EA1 | 1 | 0 | 0 | 0 | 1 | 1 | 0 | 1 | 1 | 1 | 1 | 1 | 1 |
| SRR20666376 | PRJNA862761 | SAMN29993579 | S_Typhi_QIB-NM142 | 2018 | Harare | Thilliez et al 2023 http://doi.org/10.1016/s2666-5247(23)00214-8 | 4.3.1.1.EA1 | 1 | 0 | 0 | 0 | 1 | 1 | 0 | 1 | 1 | 1 | 1 | 1 | 1 |
| SRR20666377 | PRJNA862761 | SAMN29993578 | S_Typhi_QIB-NM137 | 2018 | Harare | Thilliez et al 2023 http://doi.org/10.1016/s2666-5247(23)00214-8 | 4.3.1.1.EA1 | 0 | 0 | 0 | 0 | 1 | 0 | 0 | 1 | 1 | 1 | 1 | 0 | 0 |
| SRR20666378 | PRJNA862761 | SAMN29993577 | S_Typhi_QIB-NM136 | 2018 | Harare | Thilliez et al 2023 http://doi.org/10.1016/s2666-5247(23)00214-8 | 4.3.1.1.EA1 | 0 | 0 | 0 | 0 | 1 | 0 | 0 | 1 | 1 | 1 | 1 | 0 | 0 |
| SRR20666379 | PRJNA862761 | SAMN29993576 | S_Typhi_QIB-NM133 | 2018 | Harare | Thilliez et al 2023 http://doi.org/10.1016/s2666-5247(23)00214-8 | 4.3.1.1.EA1 | 1 | 0 | 0 | 0 | 1 | 1 | 0 | 1 | 1 | 1 | 1 | 1 | 1 |
| SRR20666380 | PRJNA862761 | SAMN29993549 | S_Typhi_QIB-NM108 | 2018 | Harare | Thilliez et al 2023 http://doi.org/10.1016/s2666-5247(23)00214-8 | 4.3.1.1.EA1 | 1 | 0 | 0 | 0 | 1 | 1 | 1 | 1 | 1 | 1 | 1 | 1 | 1 |
| SRR20666383 | PRJNA862761 | SAMN29993573 | S_Typhi_QIB-NM129 | 2018 | Harare | Thilliez et al 2023 http://doi.org/10.1016/s2666-5247(23)00214-8 | 4.3.1.1.EA1 | 0 | 0 | 0 | 0 | 1 | 0 | 0 | 1 | 1 | 1 | 1 | 0 | 0 |
| SRR20666385 | PRJNA862761 | SAMN29993571 | S_Typhi_QIB-NM117 | 2018 | Harare | Thilliez et al 2023 http://doi.org/10.1016/s2666-5247(23)00214-8 | 4.3.1.1.EA1 | 0 | 0 | 0 | 0 | 1 | 0 | 0 | 1 | 1 | 1 | 1 | 0 | 0 |
| SRR20666386 | PRJNA862761 | SAMN29993570 | S_Typhi_QIB-NM112 | 2018 | Harare | Thilliez et al 2023 http://doi.org/10.1016/s2666-5247(23)00214-8 | 4.3.1.1.EA1 | 0 | 0 | 0 | 1 | 1 | 0 | 0 | 1 | 1 | 1 | 1 | 0 | 0 |
| SRR20666387 | PRJNA862761 | SAMN29993569 | S_Typhi_QIB-NM111 | 2018 | Harare | Thilliez et al 2023 http://doi.org/10.1016/s2666-5247(23)00214-8 | 4.3.1.1.EA1 | 0 | 0 | 0 | 0 | 1 | 0 | 0 | 1 | 1 | 1 | 1 | 0 | 0 |
| SRR20666388 | PRJNA862761 | SAMN29993568 | S_Typhi_QIB-NM110 | 2018 | Harare | Thilliez et al 2023 http://doi.org/10.1016/s2666-5247(23)00214-8 | 4.3.1.1.EA1 | 1 | 0 | 0 | 0 | 1 | 1 | 0 | 1 | 1 | 1 | 1 | 1 | 1 |
| SRR20666389 | PRJNA862761 | SAMN29993567 | S_Typhi_QIB-NM109 | 2018 | Harare | Thilliez et al 2023 http://doi.org/10.1016/s2666-5247(23)00214-8 | 4.3.1.1.EA1 | 0 | 0 | 0 | 0 | 1 | 0 | 0 | 1 | 1 | 1 | 1 | 0 | 0 |
| SRR20666390 | PRJNA862761 | SAMN29993566 | S_Typhi_QIB-NM104 | 2018 | Harare | Thilliez et al 2023 http://doi.org/10.1016/s2666-5247(23)00214-8 | 4.3.1.1.EA1 | 1 | 0 | 0 | 0 | 1 | 1 | 0 | 1 | 1 | 1 | 1 | 1 | 1 |
| SRR20666391 | PRJNA862761 | SAMN29993548 | S_Typhi_QIB-NM106 | 2018 | Harare | Thilliez et al 2023 http://doi.org/10.1016/s2666-5247(23)00214-8 | 4.3.1.1.EA1 | 1 | 0 | 0 | 0 | 1 | 1 | 0 | 1 | 1 | 1 | 1 | 1 | 1 |
| SRR20666392 | PRJNA862761 | SAMN29993565 | S_Typhi_QIB-NM103 | 2018 | Harare | Thilliez et al 2023 http://doi.org/10.1016/s2666-5247(23)00214-8 | 4.3.1.1.EA1 | 1 | 0 | 0 | 0 | 1 | 1 | 0 | 1 | 1 | 1 | 1 | 1 | 1 |
| SRR20666394 | PRJNA862761 | SAMN29993563 | S_Typhi_QIB-NM96 | 2018 | Harare | Thilliez et al 2023 http://doi.org/10.1016/s2666-5247(23)00214-8 | 4.3.1.1.EA1 | 1 | 0 | 0 | 0 | 1 | 1 | 0 | 1 | 1 | 1 | 1 | 1 | 1 |
| SRR20666395 | PRJNA862761 | SAMN29993562 | S_Typhi_QIB-NM91 | 2018 | Harare | Thilliez et al 2023 http://doi.org/10.1016/s2666-5247(23)00214-8 | 4.3.1.1.EA1 | 1 | 0 | 0 | 0 | 1 | 1 | 0 | 1 | 1 | 1 | 1 | 1 | 1 |
| SRR20666396 | PRJNA862761 | SAMN29993561 | S_Typhi_QIB-NM90 | 2018 | Harare | Thilliez et al 2023 http://doi.org/10.1016/s2666-5247(23)00214-8 | 4.3.1.1.EA1 | 1 | 0 | 0 | 0 | 1 | 1 | 0 | 1 | 1 | 1 | 1 | 1 | 1 |
| SRR20666399 | PRJNA862761 | SAMN29993558 | S_Typhi_QIB-NM82 | 2018 | Harare | Thilliez et al 2023 http://doi.org/10.1016/s2666-5247(23)00214-8 | 4.3.1.1.EA1 | 1 | 0 | 0 | 0 | 1 | 1 | 0 | 1 | 1 | 1 | 1 | 1 | 1 |
| SRR20666400 | PRJNA862761 | SAMN29993557 | S_Typhi_QIB-NM81 | 2018 | Harare | Thilliez et al 2023 http://doi.org/10.1016/s2666-5247(23)00214-8 | 4.3.1.1.EA1 | 0 | 0 | 0 | 0 | 1 | 0 | 0 | 1 | 1 | 1 | 1 | 0 | 0 |
| SRR20666402 | PRJNA862761 | SAMN29993547 | S_Typhi_QIB-NM105 | 2018 | Harare | Thilliez et al 2023 http://doi.org/10.1016/s2666-5247(23)00214-8 | 4.3.1.1.EA1 | 1 | 0 | 0 | 0 | 1 | 1 | 0 | 1 | 1 | 1 | 1 | 1 | 1 |
| SRR20666403 | PRJNA862761 | SAMN29993546 | S_Typhi_QIB-NM100 | 2018 | Harare | Thilliez et al 2023 http://doi.org/10.1016/s2666-5247(23)00214-8 | 4.3.1.1.EA1 | 1 | 0 | 0 | 0 | 1 | 1 | 0 | 1 | 1 | 1 | 1 | 1 | 1 |
